# Cell Survival from Chemotherapy Depends on NF-κB Transcriptional Up-Regulation of Coenzyme Q Biosynthesis

**DOI:** 10.1371/journal.pone.0005301

**Published:** 2009-04-23

**Authors:** Gloria Brea-Calvo, Emilio Siendones, José A. Sánchez-Alcázar, Rafael de Cabo, Plácido Navas

**Affiliations:** 1 Centro Andaluz de Biología del Desarrollo, Universidad Pablo de Olavide-CSIC and Centre for Biomedical Research on Rare Diseases (CIBERER), ISCIII, Sevilla, Spain; 2 Laboratory of Experimental Gerontology, National Institute on Aging, National Institutes of Health, Baltimore, Maryland, United States of America; University of Las Palmas de Gran Canaria, Spain

## Abstract

**Background:**

Coenzyme Q (CoQ) is a lipophilic antioxidant that is synthesized by a mitochondrial complex integrated by at least ten nuclear encoded *COQ* gene products. CoQ increases cell survival under different stress conditions, including mitochondrial DNA (mtDNA) depletion and treatment with cancer drugs such as camptothecin (CPT). We have previously demonstrated that CPT induces CoQ biosynthesis in mammal cells.

**Methodology/Principal Findings:**

CPT activates NF-κB that binds specifically to two κB binding sites present in the 5′-flanking region of the *COQ7* gene. This binding is functional and induces both the *COQ7* expression and CoQ biosynthesis. The inhibition of NF-κB activation increases cell death and decreases both, CoQ levels and *COQ7* expression induced by CPT. In addition, using a cell line expressing very low of NF-κB, we demonstrate that CPT was incapable of enhancing enhance both CoQ biosynthesis and *COQ7* expression in these cells.

**Conclusions/Significance:**

We demonstrate here, for the first time, that a transcriptional mechanism mediated by NF-κB regulates CoQ biosynthesis. This finding contributes new data for the understanding of the regulation of the CoQ biosynthesis pathway.

## Introduction

Coenzyme Q (CoQ) is a small lipophilic molecule that transports electrons from mitochondrial respiratory chain complexes I and II, to complex III [1]. In addition, CoQ functions as a cofactor for uncoupling proteins [2] and other mitochondrial dehydrogenases [1]. CoQ mainly acts as an antioxidant and can prevent cell death under certain stress conditions, particularly in mitochondria-DNA depleted cells [3], [4]. CoQ also regulates the extracellulary induced ceramide-dependent apoptotic pathway [5]. CoQ is composed of a benzoquinone ring and a polyisoprenoid chain, derived from tyrosine and mevalonate, respectively. Its biosynthesis depends on a pathway that involves at least ten genes (COQ genes). Among them, *COQ7* is proposed to encode for a key regulatory component of a multisubunit enzyme complex [6]. However, there is no information about the precise regulation of CoQ biosynthesis pathway except that peroxisome proliferator-activated receptor alpha (PPARα) is involved [7].

We have previously shown that Campothecin (CPT) treatment increases CoQ biosynthesis rate and reported that CPT in mammals up-regulates *COQ7* [8]. Thus, the stimulation by CPT is a useful tool for deciphering transcriptional mechanisms of the regulation of the CoQ biosynthetic pathway in mammals through up-regulation of *COQ7* gene.

Camptothecin (CPT) is a cytotoxic drug widely used in cancer therapy. It is known that the main target for camptothecin is the nuclear topoisomerase I (TOP1) [9]–[11]. Double-strand DNA breaks derived from the inhibition of nuclear TOP1 are considered the main cause of apoptosis induction by CPT [11]. CPT also induces an increase of reactive oxygen species (ROS) in different cancer cell lines including H460 cells [12], [13], [14], [15], [16], [8].

There are recent reports supporting the role of oxidative stress in the induction of apoptosis by CPT and its derivatives [15], [17]. In response to topotecan, a CPT water-soluble derivative, cells activate their antioxidant defense mechanisms and a number of antioxidant enzyme activities, such as catalase, manganese-dependent superoxide dismutase (MnSOD), and glutathione peroxidase [16]. Also, the addition of catalase was able to protect cells from CPT induced apoptosis in HL-60 leukemia cells [12]. Furthermore, catalase administration to U-937 promonocytic cells also attenuated apoptosis induction by CPT and other cytotoxic drugs [18]).

NF-κB is a redox-sensitive transcription factor, which regulates antioxidant enzymes such as MnSOD encoded by the SOD2 gene. NF-κB is also activated by CPT in several cell types [19]–[22]. In fact, NF-κB activation is frequently abrogated by antioxidants [23], [24]. NF-κB has been shown to play a key role in the regulation of cell death, either as inducer or, more often, as blocker of apoptosis, depending on the cellular type and the insult [25], [26]. Thus, we have proposed that NF-κB could be one of the mediators of the cellular effects by CPT through the activation of the CoQ biosynthesis pathway.

## Results

### CoQ biosynthesis is dependent on NF-κB

Oxidative stress rises as an important activator of NF-κB that can be abrogated by antioxidants [23], [24]. We have previously demonstrated that in H460 cells, which were blocked in the CoQ biosynthesis pathway, exhibited a increased sensitivity to production of ROS and cell death induced by CPT [8].

**Figure 1:**
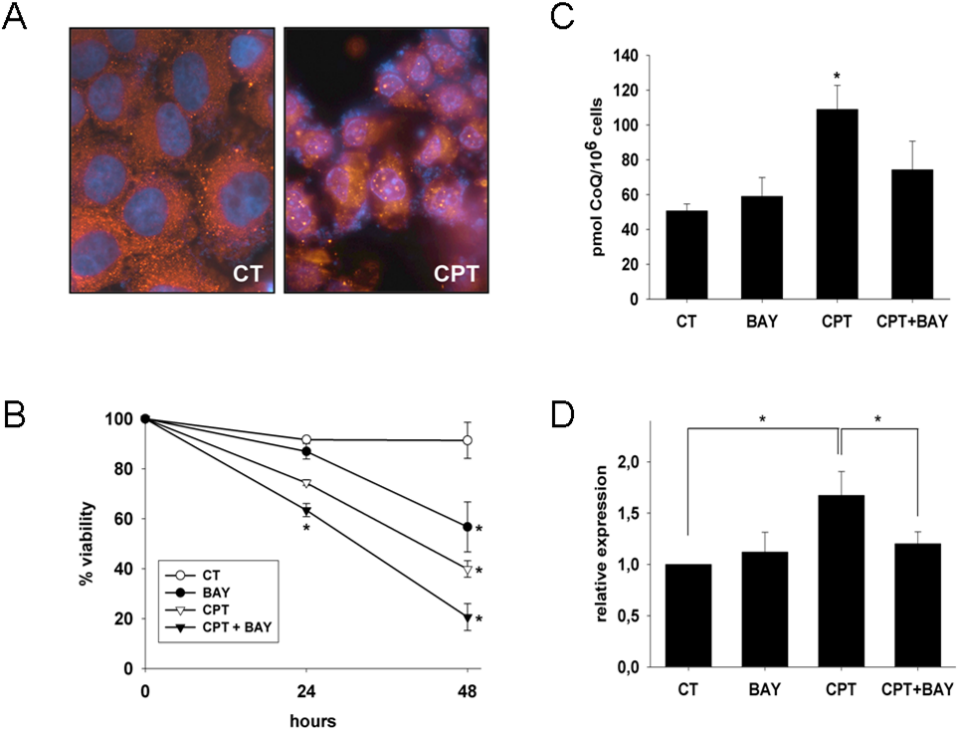
CPT activates NF-κB in H460 cells. A. H460 cells were treated, fixed and permeabilized. Immunostaining with p65 antibody shows a cytosolic distribution of the protein in control cells and a clear nuclear localization when treated with 10 μM CPT. B. Effect of the inhibitor of NF-κB on cell sensitivity to CPT. H460 viability was tested by cytometric analysis of PI incorporation. * P<0.05 between treatments and their controls C. Effect of the presence of the inhibitor Bay 11-7085 (BAY) on the CPT dependent increase in CoQ levels. Results are the mean±SD of three independent experiments. * P<0.05 between control and the CPT -treated group. D. Relative expression of *COQ7* gene measured by real time PCR. Results are normalized on the basis of the expression of the housekeeping actin gene. Results are the mean±SD of three independent experiments; * P<0.05.

H460 cells treated with 10 μM CPT for 24 hours were fixed and probed with p65 antibody to confirm that the NF-κB system is active in these cells. Immunofluorescence experiments showed that the transcription factor translocated into the nucleus in response to CPT (**figure 1 A**).

In order to test whether NF-κB elicits a survival response in H460 cells and if the induction of CoQ biosynthesis in CPT treated cells is dependent on NF-κB activation, cell viability and cellular CoQ levels were measured in the presence of Bay 11-7085, a specific inhibitor of NF-κB [27]. The inhibition of NF-κB increased cell death and reduced CoQ levels induced by CPT (**figure 1 B and C**). In parallel, we observed that the increase of *COQ7* mRNA measured by real time PCR in CPT treatments was significantly abolished by Bay 11-7085 (**figure 1 D**), suggesting a dependence of *COQ7* gene to NF-κB.

**Figure 2:**
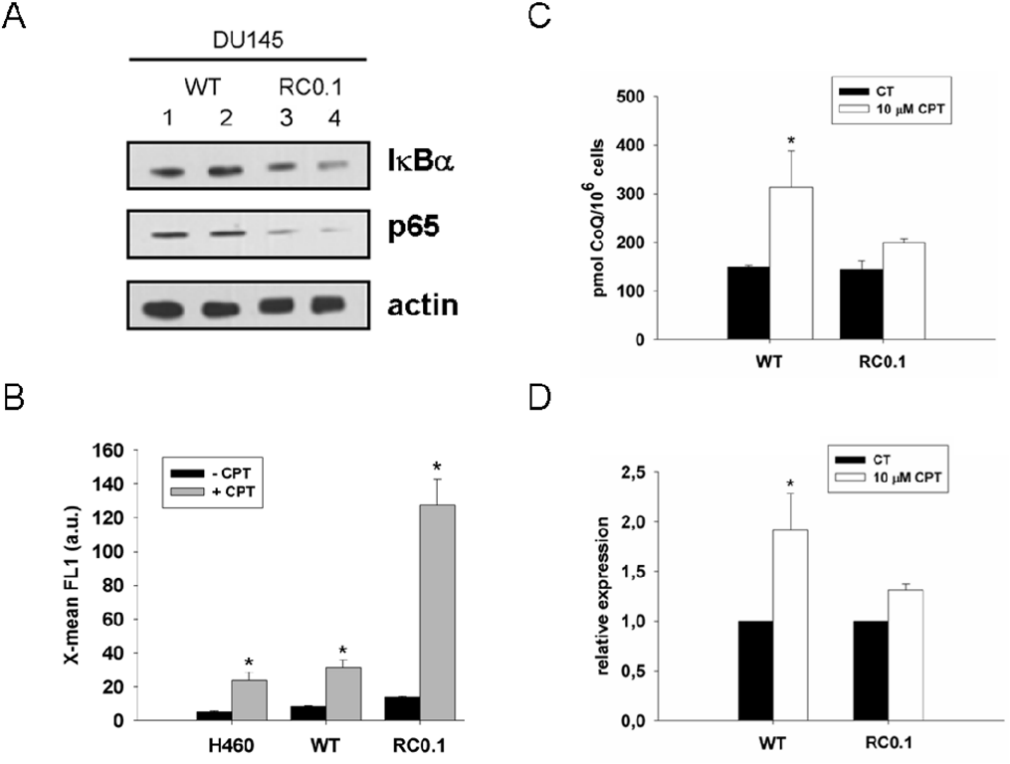
Effect of CPT on DU145 RC0.1 cells. A. Protein levels of the NF-κB system components p65 and IκBα on parental DU145 and RC0.1 cell lines. Cytosolic protein extracts from DU145WT (1 and 2) and RC0.1 (3 and 4) cells were separated on a 10% polyacrilamide gel and immunostained with antibodies against IκBα, p65 and actin. B. CPT-induced early generation of ROS. Cells were treated during the indicated times with 10 μM CPT. ROS were determined by flow cytometry by using H_2_DCF-DA. Results are expressed as mean±SD of three independent experiments; * P<0.05. C. CoQ levels of DU145 cell lines in response to 10 μM CPT. Parental DU145 and RC0.1 were treated with CPT for 24 hours and CoQ levels were determined as described under Material and Methods. * P<0.05 between control and the CPT -treated group. D. *COQ7* mRNA relative levels from parental DU145 and RC0.1 cells treated for 24 h with 10 μM CPT. Results are expressed as mean±SD of three independent experiments. * P<0.05 between control and the CPT -treated group.

It has previously been reported that NF-κB system is down-regulated in DU145 RC0.1, a cell line resistant to CPT [28]. We used parental DU145 and RC0.1 cells to evaluate protein levels of the NF-κB system components p65 and IκBα and observed a very low content in RCO.1 compared to parental cells (**figure 2 A**).

In RC0.1 cells, CPT treatment induced a higher production of ROS compared to parental DU145 and H460 cells (**figure 2 B**). Since mitochondrial ROS generation stimulated by CPT is responsible for the induction of CoQ biosynthesis, CoQ levels and *COQ7* mRNA were measured in both parental DU145 and RC0.1 cell lines after 10 μM CPT treatment for 24 h. The parental DU145 cell line showed an increase in both CoQ levels and *COQ7* messenger in response to CPT. However, the RC0.1 cells showed only a trend to increase CoQ levels and COQ7 messenger **figure 2 C and D**). Since the NF-κB system is down regulated in RC0.1 cells, this finding would confirm the requirement of NF-κB to CPT induced CoQ biosynthesis.

### NF-κB specifically binds to *COQ7* κB sites

**Figure 3:**
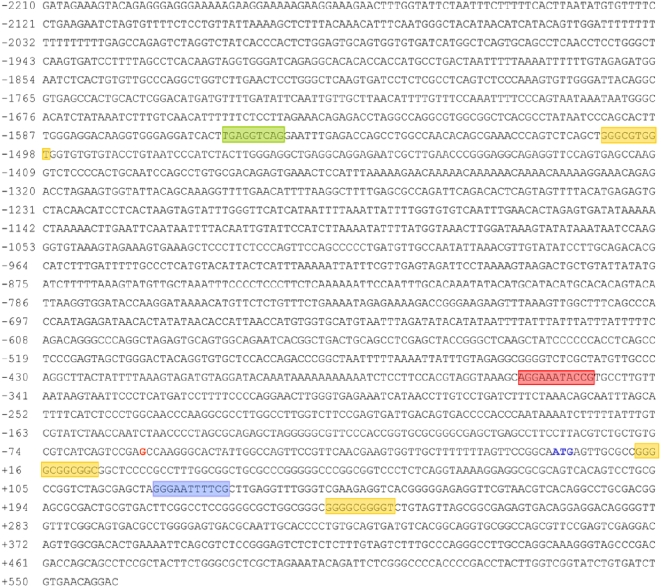
Nucleotide sequence of the 5′-flanking region of the human *COQ7* gene. Colored boxes indicate putative binding sites for diverse transcription factors: red and light blue boxes encompass κB1 and κB2 sites respectively; yellow boxes delimit hypothetical Sp1 binding sites and the green box a possible binding site for RXR. Guanine in red indicates the transcriptional start site.

Transcription element search software (TESS) sequence analysis [29], [30] of 4 Kb of the 5′-flanking region, exon 1 and the beginning of the first intron of the human *COQ7* gene revealed two potential binding sites for NF-κB, three for Sp1, as well as one for RXR (**figure 3**). The same analysis revealed other putative binding sites but we decided to focus on those potentially related to the oxidative stress response, particularly NF-κB because it is a redox-sensitive factor that has been shown to be activated by CPT [19]. One of the putative NF-κB binding sites, kB1 site, was found in the promoter region of the gene (position −360 to −350) whereas the second one, κB2, was identified at the beginning of the first intron of the gene (position +120 to +131).

**Figure 4:**
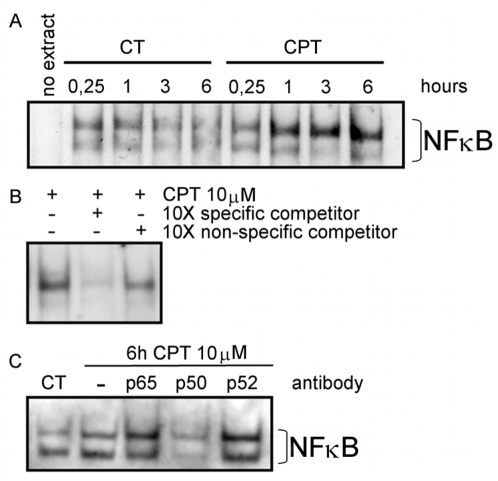
EMSA analysis of NF-κB. EMSAs were performed with H460 nuclear extracts from CPT treated and non-treated cells (CT) with digoxigenin-labeled κB1 probe. A. Time course of the NF-κB binding to the κB1 site in response to CPT. B. Competition assay using 10-fold molar concentration of unlabeled normal κB1 probe (specific) and a non related probe (non-specific) competitor. C. Antibody super shift analysis of NF-κB binding to the κB1 site in the *COQ7* promoter. Nuclear extracts from 6 hours-CPT treated H460 cells were incubated with or without different NF-κB subunits antibodies. Images are representative of various EMSAs performed.

Electrophoretic mobility shift assays were performed to evaluate the binding of the NF-κB transcription factor to the hypothetical binding sites found in *COQ7* gene. Nuclear extract from CPT treated cells were incubated with digoxigenin-labeled oligonucleotides encompassing κB1 or κB2 putative binding sites. The incubation with κB1 probe generated a band shift in response to CPT treatment (**figure 4 A**). The intensity increase of the shifted band achieved significance after 1 hour of treatment. Competition assays with an excess of either unlabeled normal or nonspecific oligonucleotides confirmed the specificity of the binding (**figure 4 B**). In addition, competition assays using antibodies against diverse subunits of NF-κB were also performed. Anti p50 impaired the formation of the protein-DNA complex. However, it failed to react with anti p65 and anti p52 sera (**figure 4 C**).

**Figure 5:**
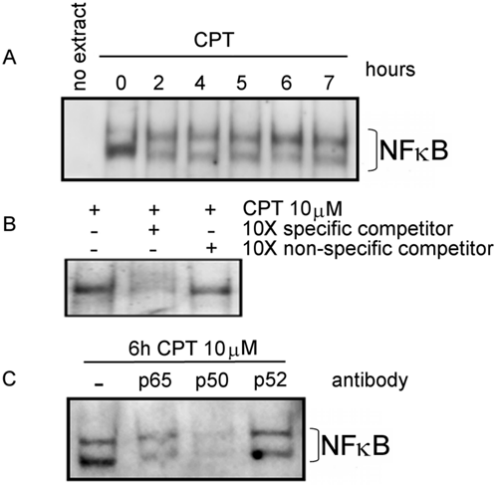
EMSA analysis of NF-κB. EMSAs were performed with H460 nuclear extracts from CPT treated and non-treated cells with digoxigenin-labeled labeled κB2 probe. A. Time course of the NF-κB binding to the κB2 site in response to CPT. B. Competition assay using 10-fold molar concentration of unlabeled normal κB2 probe (specific) and a non related probe (non-specific) competitor. C. Antibody super shift analysis of NF-κB binding to the κB2 site in the *COQ7* promoter. Nuclear extracts from 6 hours-CPT treated H460 cells were incubated with or without different NF-κB subunits antibodies. Images are representative of various EMSAs performed.

We assayed the time course binding of NF-κB to κB2 site (**figure 5 A**). Incubation of the κB2 probe with nuclear extracts of CPT treated cells resulted in a band shift that reached a maximum intensity at 6 hours of treatment. In order to demonstrate that the *in vitro* NF-κB binding to κB2 was specific and that the composition of the heterodimer was p65/p50 we ran competition (**figure 5 B**) and super-shift assays (**figure 5 C**).

Our results strongly support the *in vitro* specific binding of NF-κB to both sites in the *COQ7* sequence in response to CPT.

### *COQ7* κB binding sites are functional

**Figure 6:**
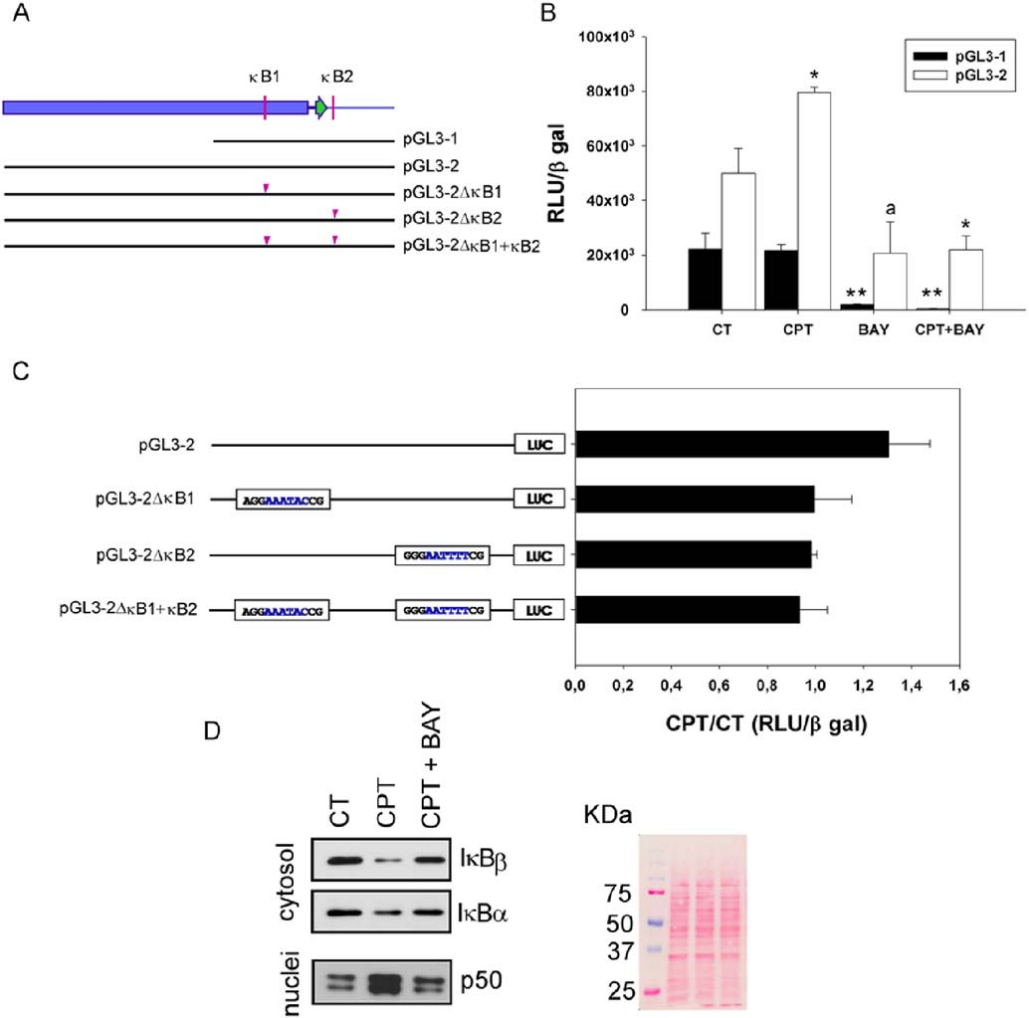
Functional analysis of the promoter of the human *COQ7* gene. A. Schematic diagram of the 5′ flanking region (blue box), 5′ UTR sequence (thick line), first exon (green arrow) and beginning of the first intron (thin line) of *COQ7* gene. Schematic illustration of the regions encompassed in the various luciferase reporter constructs used in the functional analysis. Triangles indicate deletions inside the different κB sites. B. Firefly luciferase activities expressed in HeLa cells transfected with two different reporter constructs (pGL3-1 and pGL3-2) under different treatments. Luciferase activity was normalized on the basis of the β-gal activity constitutively expressed by the co-transfected plasmid pC110. Results are the mean±SD of at least three independent transfection experiments. BAY indicates the presence of the NF-κB inhibitor Bay 11-7085. * P<0.05, ** P<0.001, a P = 0.05 between the treatment and its control. C. Deletion analysis of the *COQ7* putative κB sites. The κB sites were partially deleted as indicated in blue. CPT responsiveness of these constructs was assayed as described before. Results are expressed as the relative CPT/control response in transfected cells. Results are the mean±SD of three independent transfection experiments. D. Western blot analyzing the presence of different NF-κB system elements on the cytosolic or nuclear compartments. Punceau staining of the membrane used for the Western blot is presented as loading control.

To evaluate the physiological importance of the *COQ7* κB binding sites, firefly luciferase reporter constructs containing several regions of the *COQ7* 5′-flanking sequence (**figure 6 A**) were transfected into HeLa cells. We have previously demonstrated that CPT also induces cell death and stimulates CoQ biosynthesis in HeLa cells [8]. Transfected cells were treated with 10 μM CPT and their luciferase activity was measured. Results represent firefly luciferase activity normalized on the basis of the constitutive β-gal activity of a control vector that was co-transfected. Three hours of CPT treatment induced a significant increase in the reporter activity when pGL3-2 construct was assayed. Reporter assays performed with pGL3-1 construct, with a deletion of 1150 bp in 5′, did not show response to CPT (**figure 6 B**). These data then suggest that it is necessary a large region consisting of at least 2770 bp are required for *COQ7* to respond to CPT. The presence of the NF-κB inhibitor Bay 11-7085 during the CPT treatment of the transfected cells completely abolished the induction of the reporter gene expression (**figure 6 B**).

To further understand the system, deletions of seven bp inside the κB binding sites were made to generate the pGL3-2κB1, pGL3-2κB2 and pGL3-2κB1+κB2 plasmids. None of the constructs responded to CPT (**figure 6 C**), suggesting that both binding sites are necessary for the transcriptional induction of *COQ7* in response to CPT.

To determine which IκB isoform was involved in the NF-κB activation by CPT, cytosolic levels of both isoforms were determined by western blot. Both IκBα and IκBβ were degraded following the CPT treatment, which was prevented by Bay 11-7085. On the other hand, Bay 11-7085 also inhibited the nuclear translocation of NF-κB subunit p50 (**figure 6 D**).

## Discussion

The lipidic antioxidant CoQ is considered a central component of the antioxidant defense, protecting cells from membrane peroxidations and regenerating the reduced forms of exogenous antioxidants [31]. CoQ has also been proposed to prevent apoptosis derived from oxidative stress induced by different stimuli [32], [33].

The details about the CoQ biosynthesis pathway are unfolding. There are at least ten genes (*COQ*) involved in a complex biosynthetic pathway [34], [6]. However, there is still little knowledge about its regulation [1], except that CoQ biosynthesis is activated in mouse liver by the nuclear regulator PPARα [35]. As the number of identifiable human pathologies that are associated with a primary CoQ deficiency increases, there is a need for the full understanding of its biosynthetic pathway, from the proteins participating in the enzymatic process, to the regulatory mechanisms [36].

We have previously shown that CPT induces the up-regulation of *COQ7* [8], which is considered to have both a regulatory and kinetic role in CoQ biosynthesis [37], [6]. Thus, we have focused on the regulatory mechanisms of *COQ7* expression as a marker of CoQ biosynthetic pathway transcriptional regulation. Searching for putative binding sequences for known transcription factors we have identified two hypothetical binding sites for NF-κB through the analysis of the *COQ7* 5′ sequence using TESS program based on TRANSFACT database v6.0 [29], [30].

NF-κB activation has been observed in diverse physiological processes including immune response regulation, inflammation and development [25]. It is also active in some tumors, during chemotherapy response and under oxidative stress [38]–[40]. In fact, NF-κB activation by many agents can be abrogated by antioxidants [23], [24]. Moreover, it is known that the antioxidant enzyme MnSOD is transcriptionally activated through NF-κB in response to oxidative stress [41], [42], [21], [22]. NF-κB is generally accepted to be activated by the nuclear DNA damage exerted by CPT [19]. However, DNA damage might not be the only process by which CPT triggers NF-κB, as the antioxidant pyrrolidine dithiocarbamate (PDTC) is very effective in abolishing NF-κB activation by the drug [23].

There is no information about NF-κB involvement in CoQ biosynthesis regulation, which positively responds to oxidative stress conditions such as vitamin E withdrawal and aging [43]–[45]. Our results demonstrate that NF-κB is able to specifically bind to both *COQ7* κB sites in response to CPT. Super-shift assays indicated that the dimer that recognizes the κB2 site is composed of both p65 and p50 subunits and the homodimer p50/p50 probably binds to κB1. It is important to note that NF-κB activation by CPT has been extensively studied. However, assays have generally been done using consensus sequences corresponding to either immunoglobulin heavy chain gene [19] or the HIV enhancer [23], but we have used here a consensus sequence specific for *COQ7* gene. Thus, although it has been defined p65/p50 as the only complex involved in CPT- mediated NF-κB activation [23], we can not rule out the possibility that the *COQ7* κB2 site is recognized by a p50/p50 homodimer.

The specific binding of NF-κB to a DNA probe does not demonstrate its functionality *in vivo* but luciferase assays demonstrated that *COQ7* was transcriptionally activated by CPT. The different constructs assayed have shown that it was necessary to have at least 2150 bp of the 5′ flanking region for *COQ7* to respond to the drug. A construct containing 1000 bp of the 5′ flanking region (pGL3-1) was not able to respond to CPT. TESS analysis of the sequence of this region shows the presence of putative binding sites for Sp1 and RXR transcription factors. Interestingly, NF-κB and Sp1 have been found to cooperatively bind to DNA in different promoter systems [46]–[48]. Moreover, interaction between NF-κB and Sp1 has been also found in the promoter of the oxidative stress responsive gene *SOD2* [22]. On the other hand, RXR has been shown to be required for CoQ biosynthesis and its induction by cold exposure in mice [35].

Both basal binding and CPT-activated *COQ7* mRNA transcription promoter are inhibited by the NF-κB inhibitor Bay 11-7085, which targets the NF-κB activating IκB kinase complex (IKK) [27], supporting the NF-κB participation in the transcriptional regulation of the gene. Inhibition of NF-κB by Bay-117085 not only avoided the CPT-dependent increase of *COQ7* mRNA, but also the increase in CoQ levels. Additionally, viability assays have shown that the inhibition of the transcription factor sensitizes cells to die by CPT. All these results further support the hypothesis that CPT triggers a NF-κB survival response involving the antioxidant CoQ.

The treatment of cells with chemotherapeutic compounds such as CPT induces cell death but some cell types can survive via activation of antioxidant pathways [49], including the increase of CoQ biosynthesis [8]. Here we have shown that CPT increases ROS production. This situation activates NF-κB that, among other factors, induces the expression of *COQ7* gene by transcriptional regulation, which, in turn, increases CoQ biosynthesis. These findings contribute to the understanding of the proteins that participate in the enzymatic process of CoQ biosynthesis, which will help further our understanding of the CoQ_10_ biosynthetic pathway and its involvement in CoQ deficiency syndrome in humans.

## Materials and Methods

### Cell cultures

Non-small lung cancer cells (NSLC) line H460 was a gift from Dr. PJ Woll (CRC Department of Clinical Oncology, City Hospital, Nottingham, UK). HeLa cells were purchased from the American Type Culture Collection. DU145 WT and DU145 RC0.1 were a generous gift from Dr. Pommier (Laboratory of Molecular Pharmacology, Center of Cancer Research, National Cancer Institute/NIH Bethesda, MD). H460, parental DU145 and DU145 RC0.1 cells were cultured at 37°C in RPMI-1640 medium supplemented with L-glutamine, an antibiotic/antimycotic solution (Sigma), and 10% fetal bovine serum. HeLa cells were cultured in DMEM medium in the same conditions.

### Cell viability measurement

Dead cells become propidium iodide (PI) permeable as a consequence of the lost of plasma membrane integrity. Thus, cell viability was determined by PI (10 μg/ml) staining and flow cytometry analysis.

### Measurement of CoQ levels

Lipid extraction from cells samples was performed as described previously [8]. CoQ6 or CoQ9 were used as internal standard. Cell samples were lysed with 1% SDS and vortexed for 1 min. A mixture ethanol:isopropanol (95:5) was added and the samples were vortexed for 1 min. To recover CoQ, 5 ml of hexane was added and the samples were centrifuged at 1000×g for 5 min at 4°C. The upper phases from three extractions were recovered and dried on a rotatory evaporator. Lipid extract were suspended in 1 ml of ethanol, dried in a speed-vac and kept at −20°C until analysis. Samples were suspended in the suitable volume of ethanol prior to HPLC injection. Lipid components were separated by a Beckmann 166-126 HPLC system equipped with a 15-cm Kromasil C-18 column in a column oven set to 40 °C, with a flow rate of 1 ml/min and a mobile phase containing 65:35 methanol/n-propanol and 1.42 mM lithium perchlorate. CoQ levels were analyzed with an ultraviolet (System Gold 168), electrochemical (Coulochem III ESA) and radioactivity detector (Radioflow Detector LB 509, Berthod Technologies) when necessary.

### ROS measurement by flow cytometry

Free radical measurement was achieved by H_2_DCF-DA or CM-H_2_DCF-DA (Molecular Probes). These compounds are cell-permeant indicators for reactive oxygen species that are nonfluorescent until removal of the acetate groups by intracellular esterases and oxidation occurs within the cell. H460 cells were seeded in 35-mm dishes and treated when confluence was reached. Cells were incubated with 10 μM CM-H_2_DCFDA or H_2_DCF-DA for the last 30 min of treatment, washed and detached with trypsin. Flow cytometric analysis was performed with Epics^®^ XL cytometer (Coulter) after trypsin removal. Population was selected using FS and SS and the fluorescence was measured by FL1 detector (525±20 nm). H_2_O_2_ was used as a positive control for detection of cellular free radicals.

### Immunoblotting

Mitochondrial, nuclear or cytosolic fractions were resolved in a SDS-polyacrilamide gel electrophoresis (SDS-PAGE) (with variable acrilamide percentage according to the molecular weight of the protein of interest) and transferred to nitrocellulose membranes in a semi-dry transfer system (Trans-Blot, Biorad). After verification of equal loading using Ponceau S, membranes were blocked with 5% non-fat milk in TBS buffer (1 h room temperature or over night at 4°C) and stained with the appropriate primary antibodies (2 h at room temperature or over night at 4°C). Anti IκBα, IκBβ p50 and actin were diluted 1:1000. Incubation with anti rabbit (1:10000) or anti mouse (1:5000) HRP-conjugated secondary antibodies was performed during 2 hours at room temperature. Immunolabeled proteins were detected by membrane exposure to X-ray film after incubation in enhanced chemiluminiscence reagent (Immun-Star HRP Substrate Kit, Biorad).

### Real Time PCR

Relative expression levels were determined by real-time PCR. Cells were seeded in 35-mm plates and treated appropriately. Floating-dead cells were discarded and monolayer was washed with cold PBS. Total RNA from cells cultures was extracted with the TriPure Isolation Reagent (Roche). RNA preparation was treated for DNA removal with deoxiribonuclease I (Sigma) and cDNA was obtained from 1 μg of RNA by using the iScript cDNA Synthesis Kit (Biorad). Real Time PCR was performed in a MyiQ™ Syngle Color Real Time PCR Detection System (Biorad) coupled to a Biorad conventional thermocycler. Primers for *COQ7* and the housekeeping gene were designed with the Beacon Designer 4 software. Amplification was carried on with iQ SYBR Green supermix (Biorad) with the following thermal conditions: 30 s at 95°C and 35 cycles of 30 s at 94°C, 30 s at 60°C and 30 s at 72°C. All the results were normalized to the levels of actin mRNA. At least three independent experiments were performed and the results averaged.

### Electrophoretic mobility shift assay (EMSA)

Synthetic oligonucleotides encompassing the putative κB sites of the promoter region of the human *COQ7* gene (κB1F- TAAAGCAGGAAATACCGTGCCT; κB1R- AGGCACGGTATTTCCTGCTTTA; κB2F-AGCTAGGGAATTTTCGCTTGA; κB2R- TCAAGCGAAAATTCCCTAGCTC) were purchased from MWG Biotech AG (Germany) as single stranded oligonucleotides. Digoxigenin labelling of the oligonucleotides was performed following the DIG Gel Shift 2nd Generation Kit protocol (Roche). Briefly, complementary forward and reverse oligonucleotides were denatured 2 min at 95°C and placed at room temperature for 30 min to generate double stranded oligonucleotides. A reaction was set up in a final volume of 20 μl in 1X reaction buffer (0.2M potassium cacodylate, 25 mM Tris-HCl, 0.25 mg/ml BSA (pH 6.6) at 25°C) containing 4 pmol of double stranded oligonucleotide, 5 mM CoCl_2_, 0.05 mM digoxigenin-ddUTP and 20 U of terminal transferase and incubated 15 min at 37°C. The reaction was stopped with 2 μl of 0.2 M EDTA (pH 8.0). Resultant labelled probe was diluted to a final concentration of 16 fmol/μl with TNE (Tris-HCl 10 mM; NaCl 100 mM; EDTA 1 mM).

Preparation of nuclear extract was performed by an adaptation of the protocol described by Schreiber and co-workers [50]. Briefly, the day before the treatment H460 cells were seeded at a density of 8 10^4^ cells/cm^2^. After the treatment, cells were directly lysed on the flask by the addition of an hypoosmotic buffer (10 mM Hepes, 10 mM KCl, 0.1 mM EDTA, 0.1 mM EGTA, 0.625% NP-40, 1 mM DTT, 0.5 mM PMSF and protease inhibitor cocktail (Sigma)). Cell lysate was incubated for 5 min on ice, vigorously vortexed during 10 s and centrifuged at maximum speed for 30 s. Supernatant was reserved as cytoplasmic extract and stored at −80°C. The pellet was resuspended in an hyperosmotic buffer (20 mM Hepes, 0.4M NaCl, 1 mM EDTA, 1 mM EGTA, 1 mM DTT, 1 mM PMSF and protease inhibitor cocktail (Sigma)) and incubated on ice during 15 min in agitation. Extracts were centrifuged at 16000 g 30 min at 4°C and supernatants were recovered as nuclear protein extracts without DNA.

Electrophoretic mobility shift assays (EMSAs) were performed following the DIG Gel Shift 2nd Generation kit (Roche) indications: 20 μg of nuclear extract were incubated in 1X Binding Buffer (20 mM Hepes pH 7.6, 1 mM EDTA, 10 mM (NH_4_)SO_4_, 1 mM DTT, 0.2% Tween-20, 30 mM KCl), 1 μg poly [d(I-C)], 0.1 μg poly L-lysine and 64 fmol of DIG-labelled probe. Competition assays were performed in the presence of a 10X excess of cold specific or non specific (Oct2A: Forward strand GTACGGAGTATCCAGCTCCGTAGCATGCAAATCCTCTGG; Reverse strand CCTCATAGGTCGAGGCATCGTACGTTTAGGAGACCAGCT) probe. For supershift assays 0.4 μg of rabbit polyclonal antibody (p65, p50 or p52) was pre-incubated with the nuclear extracts for 30 min on ice before the binding incubation. Samples were loaded in a 4–8% native acrilamide: bisarilamide (30:1) gel (0.5X TBE buffer) and electrophoresed at 150 V in 0.5X TBE buffer. Electrotransference to a nitrocellulose membrane (Hybond™–N+, Amersham) was performed with a Trans-Blot Semi-Dry system (Biorad) following the manufacturer's instructions. Samples were fixed to the membrane by UV-crosslinking (70 mJ/cm^2^). Dig-labelled probes were detected with anti-digoxigenin and visualized with the chemiluminescent substrate CSPD from the DIG Gel Shift 2nd Generation Kit (Roche) and the membrane exposure to an X-ray film.

### Cloning of *COQ7* promoter region and generation of luciferase reporter constructs

Inserts in pGL3-1 and pGL3-2 vectors were generated by PCR (special thermal conditions: 6 cycles with an annealing temperature of 42°C, followed by a Touch Down-PCR starting at an annealing temperature of 62°C and finishing at 42°C) from the BAC clone RP11-626G11 (AC099518; BACPAC Resource Centre, Children's Hospital Oakland Research Institute). *COQ7* promoter region was first cloned into pGEM-T Easy Vector (Promega) using primers containing the restriction enzyme sites for SacI (5′) and HindIII (3′): hCQ7pSacIF (GTAGAGCTCTCCAAGGGTGTAA), CQ7pHindIIIR (GTTAAGCTTGTCCTGTTCACAG), for pGL3-1, and hCQ7pSacI3 (GTAGAGCTCACAGAGGGAGG) and CQ7pHindIIIR for pGL3-2. Primers were designed to amplify the 5′ flanking region of the *COQ7* gene (1000 pb for pGL3-1 and 2150 for pGL3-2), the 5′ UTR region (60 bp), the complete first exon (73 bp) and, partially, the first intron of the gene (487 bp). Once verified by sequencing, fragments were subcloned into the pGL3basic vector (a generous gift from Dr. Blesa, Centro de Investigación Príncipe Felipe, Valencia, Spain). Simple and double κB sites deletions for the pGL3-2ΔκB1, pGL3-2ΔκB2 and pGL3-2ΔκB1+2 vectors were carried out by using the Quikchange II XL Site-Directed Mutagenesis Kit (Stratagene) with the following primers pairs: for κB1 site, CCTTCCACGTAGGTAAAGCAGCGTGCCTTGTTAATAAGTAAT and ATTACTTATTAACAAGGCACGCTGCTTTACCTACGTGGAAGG; for κB2 site CGGTCTAGCGAGCTAGGCGCTTGAGGTTTGGGTC and ACCCAAACCTCAAGCGCCTAGCTCGCTAGACCG.

### Reporter gene assays

Endotoxin-free plasmids were obtained by Qiagen EndoFree® Plasmid Maxi Kit, to avoid unspecific activation of NF-κB. HeLa cells were transfected with FuGENE 6 (Roche) following the manufacturer's instructions. Briefly, the day before transfection, 2.9×10^5^ cells were plated in 12 well dishes (DMEM 10% serum without antibiotics). One hour before transfection, media was changed. For each single transfection 2.2 μl of FuGENE reagent were diluted in 50 μl of non supplemented OPTIMEM media (Gibco) and incubated for 10 minutes at room temperature. 0.74 μg of total DNA was added to the FuGENE diluted transfection mixture and further incubated for 30 minutes at room temperature. Then, the transfection reagent/DNA complex was added to the cells. Cells were co-transfected with pGL3-1 or pGL3-2 (0.37 μg) and pCH110 (0.37 μg), a plasmid containing the β-galactosidase gene regulated by a constitutive promoter (a generous gift from Dr. Carrión, Universidad Pablo de Olavide, Sevilla, Spain). The transfection proceeded for 48 hours before cells were appropriately treated. After that, death cells were discarded and monolayer was washed with cold PBS and lysed in 100 μl of Luciferase Lysis Buffer (Promega). Lysates were vigorously vortexed for 10 s and centrifuged 12000 g during 15 min. Aliquots were stored at −80°C until used. β-galactosidase activity was quantified in the same extracts used for luciferase activity measurement. 30 μl of lysate sample was mixed with 20 μl of H_2_O and 50 μl of reaction buffer (200 mM Na_2_HPO_4_ pH 7.3, 2 mM MgCl_2_, 100 mMβ-mercaptoethanol, 1.33 mg/ml ONPG [o-nitrophenyl-β-D-galactopyranoside]). Samples were incubated for 45 min/ 1 h at 37°C and β-galactosidase activity was spectrophotometrically measured at 405 nm in a Sunrise plate reader (Tecan Austria GmbH) coupled to the Magellan software. Luciferase activity was measured by a manual luminometer Luminoskan TL Plus (Thermo LabSystems). 2 μl of cell extract were added to 50 μl of reaction buffer (Luciferase Assay Buffer from Promega Luciferase Assay System kit) in a luminometer cuvette. Light intensity was measured inside the detection linear range. Results are expressed as relative intensity units (LRU). Definitive results are expressed as relative LRU/ β-gal activities.
